# Epigenetic signatures of invasive status in populations of marine invertebrates

**DOI:** 10.1038/srep42193

**Published:** 2017-02-16

**Authors:** Alba Ardura, Anastasija Zaiko, Paloma Morán, Serge Planes, Eva Garcia-Vazquez

**Affiliations:** 1 PSL Research University: EPHE-UPVD-CNRS, USR 3278 CRIOBE, Université de Perpignan, 52 Avenue Paul Alduy, 66860 Perpignan Cedex, France; 2 Laboratoire d’Excellence “Corail”, Centre de Recherche Insulaire et Observatoire de l’Environment (CRIOBE), BP 1013, 98 729 Papetoai, Moorea, French Polynesia; 3 Coastal and Freshwater Group, Cawthron Institute, 98 Halifax Street East, 7010 Nelson, New Zealand; 4 Marine Science and Technology Centre, Klaipeda University, H. Manto 84, LT-92294, Klaipeda, Lithuania; 5 Facultad de Biología, University of Vigo. Campus Universitario Lagoas-Marcosende, 36310 Vigo, Spain; 6 Department of Functional Biology, University of Oviedo. C/Julian Claveria s/n 33006-Oviedo, Spain

## Abstract

Epigenetics, as a DNA signature that affects gene expression and enables rapid reaction of an organism to environmental changes, is likely involved in the process of biological invasions. DNA methylation is an epigenetic mechanism common to plants and animals for regulating gene expression. In this study we show, for the first time in any marine species, significant reduction of global methylation levels during the expansive phase of a pygmy mussel (*Xenostrobus securis*) recent invasion in Europe (two-year old), while in older introductions such epigenetic signature of invasion was progressively reduced. Decreased methylation was interpreted as a rapid way of increasing phenotypic plasticity that would help invasive populations to thrive. This epigenetic signature of early invasion was stronger than the expected environmental signature of environmental stress in younger populations sampled from ports, otherwise detected in a much older population (>90 year old) of the also invasive tubeworm *Ficopomatus enigmaticus* established in similar locations. Higher epigenetic than genetic diversity found in *X. securis* was confirmed from *F. enigmaticus* samples. As reported for introduced plants and vertebrates, epigenetic variation could compensate for relatively lower genetic variation caused by founder effects. These phenomena were compared with epigenetic mechanisms involved in metastasis, as parallel processes of community (biological invasion) and organism (cancer) invasions.

Epigenetics, particularly a noticeable shift in methylation status, is often associated with the process of colonization of new environments. This is a natural response to changes in abiotic factors^1^ or biotic environment^2^. Changes in methylation are thought to be involved in phenotypic plasticity^3^, that is an important prerequisite for adaptation to varying environmental conditions reported both in plants and animals^1^,^4^. Different types of stressing environmental conditions may alter global methylation levels^5^,^6^,^7^,^8^. The direction of the change in global methylation may depend on particular stressors; for example, some chemicals present in environment may reduce methylation while others tend to increase it^5^,^9^. A few *in situ* studies reported hypermethylation response in aquatic animals exposed to environmental stress^6^,^10^.

Due to its importance in adaptation mechanisms, epigenetics must be involved in the process of biological invasions facilitating the establishment of exotic organisms in recipient ecosystems^11^,^12^. The epigenetic variation in introduced populations may to a certain level compensate for reduced genetic diversity and serve as an alternative source of phenotypic variation. Evidences of epigenetic, not genetic, response to environmental variance in invaded habitats and therefore differentiation of populations are reported for introduced plants and birds^13^,^14^,^15^. These findings can add a new perspective to the understanding of biological invasions and underlying mechanisms^16^. Plasticity or new phenotypes acquired through epigenetic changes can explain the ability of invasive species to expand and colonize new ecosystems with very reduced genetic diversity due to founder effects^17^. Some invasive populations exhibit considerable genetic diversity resulting from repeated introduction events^11^,^18^, and have therefore a sufficient substrate of genetic variants for selection and response to different environmental conditions. However, epigenetic mechanisms provide a faster way of response because they may occur in one generation, while selection implies differential reproduction of genetic variants – so at least two generations are needed. Hence, successful invaders are expected to be prone to epigenetic variations and this might be reflected in their epigenetic signatures regardless the genetic diversity of the invasive population.

To date, the available data about invasion epigenetics refer predominantly to terrestrial species. The epigenetics of marine invasions is largely understudied yet. Biological invasions follow a sequential process of arrival, establishment, expansion, and eventually accommodation within the recipient ecosystem^19^,^20^,^21^. The maximum alteration at epigenomic level is expected to occur at the arrival through early expansion phase, when the species needs to boost its adaptive capacity to overcome the existing environmental constraints and establish a successful population.

Here we present a proof-of-concept study aimed at challenging our hypothesis of epigenetic signature in invasive populations, based on the New Zealand pygmy mussel *Xenostrobus securis*. This species is invading the coastal waters of Japan^22^ and south Europe^23^,^24^. A new arrival was detected in 2014 in a port from northwest Iberian Peninsula (southwest Bay of Biscay), where it is expanding dramatically fast^18^,^25^,^26^. Epigenetic and genetic variation of this new population were compared with older invasive European populations from another port and a lagoon protected under NATURA 2000. Considering that: environmental stress induces methylation changes (sometimes hypermethylation but not always); some introduced populations exhibit higher epigenetic than genetic diversity; and lower methylation would encompass higher phenotypic plasticity, expectations were: I) mussels would be differentially methylated in polluted ports compared to cleaner lagoons; II) higher epigenetic than genetic diversity would be expected in invasive mussels if the process described for vertebrates and plants is common to all invasions, that is, these introduced samples will differ more for epigenetic than for genetic variation; III) the newer population, undergoing the initial expansion phase, would exhibit lower global methylation than already established ones –i.e. for similar port environment the newer invader would be less methylated than the older one.

To check if the results may be generalizable we have also analyzed a few individuals from comparatively older introduced populations of Australian tubeworm *Ficopomatus enigmaticus* from markedly different environments. This is a worldwide well-established marine invader^27^,^28^,^29^, of very old introduction in the Bay of Biscay (back to 1921^30^). Following the same rationale, these Bay of Biscay samples would be more methylated than newer ones. Contrasting environmental conditions were also considered for the newer populations: one more recent from a big port in New Zealand and another of intermediate age from a protected Mediterranean lagoon.

## Material and Methods

### Sampling sites and environmental conditions

The *X. securis* and *F. enigmaticus* specimens analyzed in this study were all adults, collected in winter time (December 2014–January 2015 in Europe, July in New Zealand). The sampling sites represented different environments (Table 1), from lower to higher environmental stress: protected lagoons in the Mediterranean; one local fishing port in a small village (Llanes in the Bay of Biscay); commercial ports with international maritime traffic nearby industrial cities (Aviles in the Bay of Biscay, Pontevedra in Northwest Spain, Napier in New Zealand). General environmental information for the sampling areas was collected from the online resources (e.g. National Agency of Meteorology, Global Sea Temperature website, ClimaTemps website, National Ports –Spanish ports at http://www.puertos.es/es-es, Napier port at http://www.napierport.co.nz/), published national and regional reports and research papers. The date of the first record of the species was verified with the published literature resources and on-line databases (Invasive Species Specialist Group, IUCN; WRIMS; AquaNIS^31^).

### Ethical statement

This study has been carried out on invertebrate invasive species, thus measures of careful cleaning and disinfection of materials and clothes after sampling were taken, to avoid further dispersion of these organisms. This study adheres to the European Code of Conduct for Responsible Research.

### Sample collection, DNA extraction and barcoding

The two species here studied have a short planktonic larval stage, are tolerant to wide salinity, temperature and pollution ranges and can disperse through shipping pathway^27^,^32^. Adult individuals of *X. securis* (25, 26 and 31 from Mediterranean, Atlantic and Cantabric respectively) were identified *de visu* and preserved in ethanol for further genetic and epigenetic analysis. Total DNA was extracted from a small piece of foot muscle with the E.Z.N.A Mollusc DNA kit (IOMEGA, bio-tek), following manufacturer´s instructions. Five individuals of *F. enigmaticus* were sampled from each site and preserved in ethanol as well. DNA was extracted from the whole body employing a method based on silica gel columns (QIAmp DNA Mini Kit, Qiagen), following manufacturer’s instructions.

The tubes with DNA samples were stored at 4 °C for immediate analysis and aliquots were frozen at −20 °C for long-time preservation. Isolated DNA was quantified using a fluorometric method with Qubit^®^ 2.0, and normalized to 100 ng/μ for subsequent analysis.

DNA barcoding was performed for each individual to verify the identity of the species and for the further reference. The mitochondrial cytochrome c oxidase subunit I (COI) gene was amplified from *X. securis* samples using the universal primers designed by Geller *et al*.^33^ and the conditions described therein. The nuclear subunit 18 S rRNA gene was PCR amplified from *F. enigmaticus* due to the absence of COI gene references in publically available databases (BOLD Systems, NCBI) at the moment of this study (June, 2016), using the primers and protocol described in Distel *et al*.^34^. Bovine serum albumin (BSA, 200 ng/μl) was included in the PCR protocol described by Distel *et al*.^34^ to avoid interferences of possible inhibitors.

PCR products were examined on 2% agarose gel stained with SimplySafeTM (EURx, Poland). Positive amplicons (evidenced by clear single band of the expected size) were sequenced by Macrogen Inc. (The Netherlands) with ABI3730xl DNA sequencer (Applied Biosystems).

Obtained DNA sequences were edited with BioEdit v7.2.5^35^ and compared with those published in online databases using nBLAST tool (www.ncbi.nlm.nih.gov/).

### MSAP analysis

The methylation-sensitive amplified polymorphism approach or MSAP was used to detect polymorphism in DNA methylation patterns. The protocol for global methylation analysis was conducted following Díaz-Freije *et al*.^36^. An aliquot (100 ng) of DNA of each sample was split in two parts to be treated with either *EcoRI*/*HpaII* or *EcoRI/MspI.* Both enzymes (*MspI* and *HpaII*) recognize and cleave CCGG target sequences, but cleaving by *HpaII* is blocked when the inner or outer C is methylated at both strands; while cleaving in *MspI* is blocked when the outer cytosines are fully or hemi-methylated; cleaving in both enzymes is blocked when both cytosines are methylated –and/or when nucleotide polymorphism occurs in the restriction target thus the sequence is not recognized by the enzymes^37^.

The resulting DNA fragments were ligated with linkers and PCR amplified using two primer combination: *EcoRI*-AAG, *HpaII*-TCC and *EcoRI*-AAG, *HpaII*-TAC. *HpaII* primers were end-labeled using 6-FAM reporter molecule^38^. PCR products were loaded with a GeneScan GS-500 LIZ3130 size standard into an ABI Prism 3100 Genetic Analyzer (Applied Biosystem). Fragment analysis and AFLP scoring was performed using GeneMapper v.4.0 software (Applied Biosystem). To avoid confounding methylation sites and poorly reproducible fragments the following settings were applied: analysis range, 50–500 base pairs (bp); minimum peak height, 50 relative fluorescence units; pass range for sizing quality: 0.75–1.0; maximum peak width: 1.5 bp; maximum peak height ratio: 1.8 (higher peaks were removed); normalization method: sum of signals. To confirm AFLP reproducibility the five *F. enigmaticus* and five *X. securis* samples (XAv1–5) from the Bay of Biscay were analyzed again with the same protocols.

### Data analysis

MSAP individual and population profiles were analyzed using the R package msap v.3.2.2.^39^. The software combines the information based on the four possible patterns from presence-absence matrices obtained with the *EcoRI-HpaII* and *EcoRI-MspI* primer combinations, yielding a new score matrix according to the methylation state. The type of epigenetic variation detected with MSAP loci was categorized following Salmon *et al*.^37^:

-*Type I* = restriction site no methylation: both enzymes cut at the restriction site,

-*Type II* = methylation of internal C: HpaII does not cut and MspI does cut,

-*Type III* = methylation of external C or hemimethylation: HpaII does cut and MspI does not, and

-*Type IV* = hypermethylation or mutation in restriction site: neither enzymes cut.

The study developed by Fulnecek and Kovarik^40^ indicated that type II and III variation cannot be interpreted as CG versus CGH methylation, because what looks like CHG methylation is in fact often caused by differently methylated internal restriction sites nested with fragments. Type IV variation is not employed for calculating methylation state because it could be also due to mutations in restriction sites, so methylation state cannot be specified^37^. Therefore, we pooled data in two categories, methylated (Type II and III) or not methylated (Type I) restriction sites. The global methylation level was thus measured following Nicotra *et al*.^41^ as the proportion of methylated loci (Types II and III) over the scorable loci (Types I, II and III), per dataset.

Every locus was classified as either Methylation-susceptible loci (MSL) or Non-methylated loci (NML), depending on whether the observed proportion of methylated states across all samples exceeded a user-defined error rate-based threshold (ERT; 5% by default). Only those fragments showing polymorphism, with at least two occurrences of each state, were used for subsequent analysis^42^. MSL were used to assess epigenetic variation and NML were analyzed in order to asses genetic variation among populations as their banding pattern depends exclusively on changes in the sequence at the restriction target.

The following analyses were performed in MSAP using the R package msap v.3.2.2.^39^, for both, MSL and NML. The amount of overall variation was estimated using the Shannon diversity index (I). Differences between Shannon’s indices between MSL and NML were tested using the Wilcoxon rank sum test with continuity correction (W).

The epigenetic (MSL) and genetic (NML) differentiation among populations and between pairs of populations was assessed by means of ɸ_ST_ values (equivalent to F_ST_ values in codominant loci), and principal coordinates analyses (PCoA) followed by analysis of molecular variance (AMOVA)^43^, using the R package msap v.3.2.2.^39^ and GenAlEx software^44^,^45^.

The mean proportion of methylated loci was compared among populations using classic ANOVA. Analysis of residuals was done and normality was checked with Shapiro-Wilk test; if it was significant or not interpretable Welch F test was performed instead of ANOVA. In that case clear outlier data were removed for pairwise analyses. Medians were compared among populations with Kruskal-Wallis test. Post-hoc pairwise comparisons were made with Tukey’s honest significance tests for means, and Mann-Whitney for medians. Software PAST^46^ was employed to perform these statistical tests.

Finally, genetic differences between pairs of populations were also assessed by comparing *EcoRI-HpaII* and *EcoRI-MspI* profiles as standard AFLPs using the option meth(false) implemented in the R package msap. This is a second measure of genetic variation that scores all the loci, not only NML. Consistent results for the two measures would reinforce the conclusions about genetic differences between populations. For confirmation ɸ_ST_ values were also obtained and AMOVA performed for population pairs using GenAlEx software^44^,^45^.

## Results

In total 82 *X. securis* and 15 *F. enigmaticus* adults, from three different introduced populations for each species, were barcoded for species confirmation and analysed for AFLP and MS-AFLP variation. The most frequent haplotypes of COI and 18 S rRNA gene sequences obtained from the analyzed *X. securis* and *F. enigmaticus* individuals (648 and 656 nucleotides respectively), are available in NCBI GenBank database with the accession numbers KX129960-KX129962 and KX129957-KX129959. Comparison of the acquired sequences with existing references in nucleotide databases confirmed unambiguously the species identity of the individuals analyzed.

Detected AFLP variation was considerable in the two species. In total 380 AFLP loci were found in the *X. securis* samples (Supplementary Table 1 with Dataset 1) and 188 in *F. enigmaticus* (Supplementary Table 2 with Dataset 2). Of those, 200 (52.63%) and 105 (55.6%) were methylation-susceptible loci (MSL) in *X. securis* and *F. enigmaticus* respectively. The results were reproducible because the 5 individuals reanalyzed of each species gave the same AFLP and methylation patterns (data not shown). The genetic differentiation based on all the AFLP loci provided higher statistical significance for pairwise ɸ_ST_, as expected from the higher number of loci examined and distant populations of likely multiple origin^18^. Indeed significant differences were detected between all pairs of *X. securis* populations (data not shown). Significant differences occurred between *F. enigmaticus* FMed and FNZ samples (ɸ_ST_** = **0.082, P = 0.018), but not between FAtl and FMed (ɸ_ST_** = **0.015, P = 0.249), neither between FCant and FNZ (ɸ_ST_** = **0.0501, P = 0.061), probably due to small sample sizes.

### Xenostrobus securis

**Figure 1:**
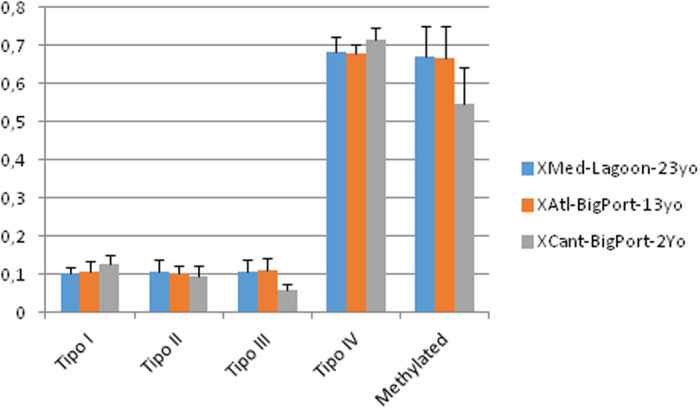
DNA methylation in methylation-sensitive loci detected from all analyzed specimens of *Xenostrobus securis*, per population. Type I to IV are respectively: no methylated, methylation of internal C, methylation of external C or hemimethylation, and hypermethylation or mutation in restriction site. Methylated: Global methylation level estimated following Nicotra *et al*. (Nicotra *et al*.^41^), as proportion of (Type II+Type III loci)/(scorable loci). The letters yo mean “year old”.

There was considerable variation in MSL between individuals within each population. The proportional occurrence of each type of methylation for *X. securis* from different locations is summarized in Fig. 1. All of the samples exhibited more fully methylated –or mutations in restriction sites- (Type IV) than hemimethylated (Type III), internally methylated (Type II) and unmethylated loci (Type I).

For the individual number of methylated loci in the analyzed mussels, Shapiro-Wilk test (value of 1) was not statistically significant, meaning the distribution did not deviate significantly from normality. The mean proportion of methylated MSL loci exhibited strong statistical significance among groups (F = 18.34, P ≪ 0.0001) and the same occurred for medians (Kruskal-Wallis test with P ≪ 0.0001; Table 2). The pygmy mussel population recently introduced into a big port, XCant, was hypomethylated in comparison with the other two populations of this species analyzed. The difference between this sample and the other two was statistically highly significant for both means and medians, as indicated from pairwise Tukey’s and Mann-Whitney tests (Table 2). Methylated loci (Types II and III) represented 55% of the total scorable loci (Fig. 1, column at right). In contrast the other two populations were quite similar to each other, with 67% methylated loci in both population: the relatively young population from the other big port (XAtl) and the older population introduced in a Mediterranean lagoon (XMed), without statistical differences between them (Table 2).

On the other hand, global epigenetic differences (i.e. in MSL) were detected among *X. securis* populations (highly significant AMOVA, **ɸ**_**ST**_ = 0.1447, P < 0.0001). From a total variance of 42.323, 14.47% was due to variation among populations. For genetic variation (NML) the populations were also globally significantly different (**ɸ**_**ST**_ = 0.1029, P < 0.0001), but the variance due to among-population variation was lower, 10.29% of the total variance (1.345 for a total variance of 13.065). Epigenetic diversity (at MSL) was indeed significantly higher than genetic diversity (NML), Shannon’s index being I = 0.5754 (SD = 0.1189) and I = 0.2510 (SD = 0.1483) respectively, W = 31834 (P < 0.0001).

**Figure 2:**
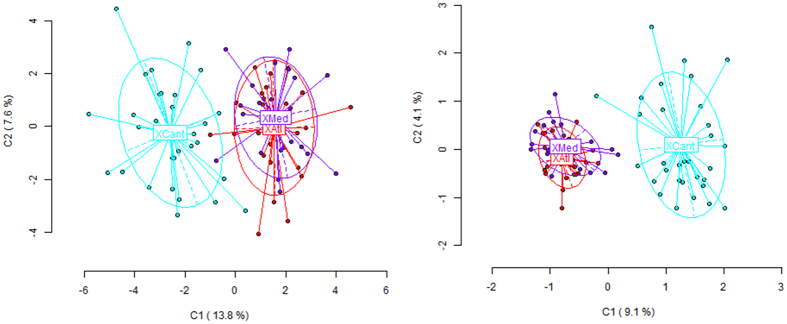
Two-dimensional visualization of the Principal Component Analysis (PCoA) of the detected methylation patterns in *Xenostrobus securis*, with the epigenetic variation (methylation-sensitive loci) on the left and the genetic variation (no methylated loci, NML) on the right. The individuals of each population are represented by the acronyms XAtl, XCant and XMed for the Atlantic international port, Cantabric international port and Mediterranean lagoon populations respectively.

The difference between epigenetic and genetic variation in *X. securis* dataset could be visualized from the PCoA (Fig. 2). The methylation patterns (MSL, Fig. 2, left) reported for *X. securis* from the Mediterranean lagoon (XMed) and Atlantic international port (XAtl) samples overlapped partially, with the new expansive population from the Bay of Biscay port (XCant) clearly differentiated. For NML (Fig. 2, right) the variation was lower. Pairwise differences for MSL involving XCant were highly significant (Table 3, below diagonal). XMed (23 year-old population) was expected to be less methylated than XAtl (13 year old) because of less disturbed environment, but it was slightly more methylated –although not significantly (**ɸ**_**ST**_ = 0.012, P = 0.1567 for MSL). Despite not significantly difference for epigenetic variation, these two populations did differ significantly for their genetic variation (**ɸ**_**ST**_ = 0.016, P = 0.0035 for NML; Table 3 above diagonal).

### Ficopomatus enigmaticus

**Figure 3:**
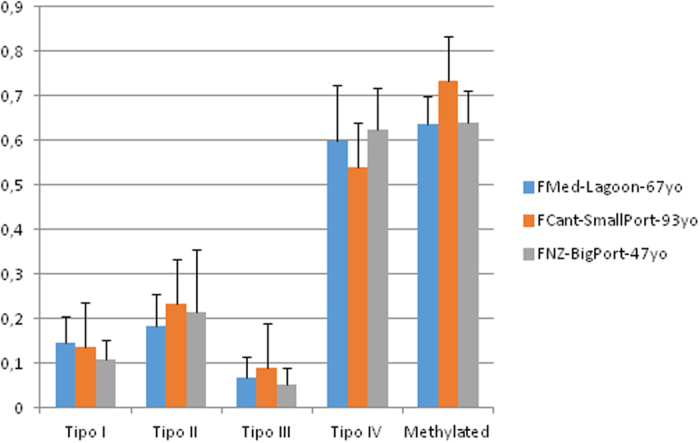
DNA methylation in methylation-sensitive loci detected from the analyzed specimens of *Ficopomatus enigmaticus*. Type I to IV are respectively: no methylated, methylation of internal C, methylation of external C or hemimethylation, and hypermethylation or mutation in restriction site. Methylated: Global methylation level estimated following Nicotra *et al*.^41^, as proportion of (Type II+Type III loci)/(scorable loci). The letters yo mean “year old”.

The tubeworm *Ficopomatus enigmaticus* individuals examined were also variable for AFLP, with 105 MSL (50% polymorphic) and 83 NML (99% polymorphic). As in the case of *X. securis,* more fully methylated –or mutation in restriction site- (Type IV) than hemimethylated (Type III), internally methylated (Type II) and unmethylated loci (Type I) were detected (Fig. 3). The global methylation level was higher in the long-time established samples of the Bay of Biscay fishing port (FCant, 73%) than in the Mediterranean lagoon and Napier port (63.5% and 63.9% respectively) (Fig. 3, columns “Methylated”, on the right-side). The Shapiro-Wilk test provided a NaN value of 1.989 for the individual proportion of methylated loci in the 15 samples analyzed. The Welch F test for samples with unequal variances was marginally significant (F = 4.969, P = 0.052). Differences among medians measured from Kruskal-Wallis test were not significant but not far from significance (P = 0.067) (Table 2). After removing the two clearly divergent individuals FCant5 and FNZ1, and despite small sample sizes, the difference between FCant and the other two samples was nearly significant for the Mann-Whitney test, although not for the post-hoc Tukey’s test (Table 2).

**Figure 4:**
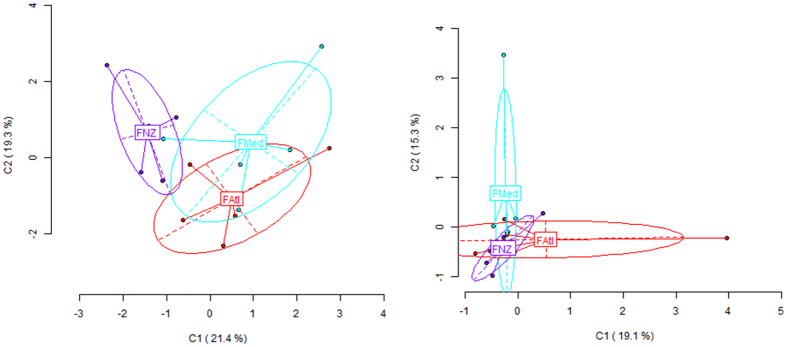
Two-dimensional visualization of the Principal Component Analysis (PCoA) of the detected methylation patterns in *Ficopomatus enigmaticus*, with the epigenetic variation (methylation-sensitive loci) on the left and the genetic variation (no methylated loci, NML) on the right. Each population is represented by the acronyms FNZ, FCant and FMed for samples, respectively, from the international Napier port in New Zealand, Cantabric fishing port and Mediterranean lagoon locations.

As it happened in *X. securis*, higher variation was found for MSL (epigenetic) than for NML (genetic). The average Shannon’s diversity indices were I = 0.5869 (SD: 0.1055) and I = 0.2843 (SD: 0.0873) for MSL and NML respectively, significantly different according to Wilcoxon signed rank test (W = 4206, P < 0.0001). According to lower genetic than epigenetic diversity, the PCoA for this species (Fig. 4) showed partial population overlapping for MSL (Fig. 4, left) and clearly lower variation with higher population overlaps for NML (Fig. 4, right).

The three population samples analyzed did not exhibit significant genetic differences among them based on the 83 NML loci examined (ɸ_ST_ = 0.0079, P = 0.375; variance among and within populations being 0.053 and 6.733 respectively). Accordingly, significant difference did not occur between any pair of samples (Table 3, above diagonal). In contrast with NML, the epigenetic differences (for MSL) among the analyzed samples were highly significant (ɸ_ST_ = 0.1654, P = 0.0018, for variances among and within populations of 1.88, 9.488). The pairwise differences at MSL between the more recently introduced Napier international port population (FNZ) and the other two samples were significantly different (Table 3, below diagonal). The difference between the Mediterranean lagoon (FMed) and the Bay of Biscay port (FCant) samples was not significant (ɸ_ST_ = 0.021, P = 0.3428).

## Discussion

Our study is the first reporting changes in methylation patterns of invertebrates likely associated with the process of biological invasions. In invasive *X. securis* introduced to ports of similar size and pollution levels, significant hypomethylation was found in the most recent population XCant versus the 13-year older XAtl. This latter, also under high anthropogenic pressure, and an older population under lesser stress in a Natura 2000 lagoon (XMed) were similarly methylated. These populations (XAtl and XMed), with a significant differentiation in genetic variation and their very different stress level, were expected to be significantly different for global methylation^4^,^9^. The results however reported the opposite. *X. securis* is likely under expansion in the northwest Iberia sampling region^24^, and, although XMed was older than XAtl, its methylation level probably represented still the “invasive” signature of the species rather than its response to environmental stress. All this suggests that the hypomethylated signature of early invasions may override the expected environmental signature in the newer populations.

Despite limited sample sizes for *F. enigmaticus* analysis, the results pointed to the same direction. For this species the older population, sampled from a small fishing port, seemed to be more methylated than populations from differentially disturbed settings (an international port in New Zealand and a lagoon under Natura 2000 network protection). Although for quantitative tests (proportion of methylated loci) statistical significance of *p* < 0.05 was not reached, highly significant differences were found for ɸ_ST_ analysis between the more recent population established in the New Zealand port and the two older ones (Table 3). Assuming ports are disturbed areas, absence of differential epigenetic patterns between the older Bay of Biscay port and the intermediate Mediterranean lagoon samples would contradict the expectations of higher methylation under environmental stress in polychaetes^6^. Perhaps the epigenetic signature of (relatively) recent invasion was overriding the environmental signature also in *F. enigmaticus*, as it seemed to happen in *X. securis*. The expected influence of environmental stressors on methylation^4^,^9^, principally due to disturbed environment in big ports in this case study, would be perhaps detected in native populations without the “invasive” signature.

Taken together, the results of this study would support our departure hypothesis of relaxed methylation control in early invasions. All the individuals here analyzed were adults, thus ontogenetic methylation changes can be reasonably excluded as a reason for global methylation differences. The phenomenon of reduced methylation could be a function of the invasion phase (expanding populations versus the accommodated ones), and would have a role in invasion processes. At organism level, in vertebrates DNA methylation switches-off gene expression during the development but can also arise when ageing, perturbing the animal organism and causing disease^47^. Uncontrolled growth is generally associated with decreased global methylation in cell populations^48^,^49^. Our results could suggest a parallelism between cancers and biological invasions. In a way, the uncontrolled spread of nuisance species in an ecosystem (bioinvasion outbreak) could be compared to the uncontrolled growth of cells in an organisms (cancer). The two invertebrates studied here are extremely invasive, populations grow rapidly and exhibit incredible dispersal potential^27^,^32^. They could be compared with potentially malignant tumors that can reproduce without control. Epigenetic changes involved in malignant cells that cause tumor growth affect some groups of specific loci, that are generally hypermethylated^50^. If the process advances and the malignant cells move to other tissues (metastasis) new epigenetic phenomena appear. Global hypomethylation coupled with hypermethylation of some specific DNA regions are typical signatures of some metastatic cancers^51^,^52^. From our modest but significant results, in recent invasive populations undergoing expansive growth (like our *X. securis* case) even adult individuals would exhibit a global signature of decreased methylation - compared to those from earlier established populations.

The clear understanding of epigenetic contribution to the species invasiveness is difficult from observational studies. There is poor knowledge on the current status of the local invasive populations (their abundance and distribution range, magnitude of environmental impacts) and the details of the invasion history (date of introduction, establishment, number and sources of repeated incursions, etc.). Yet, in the present case there is no doubt XCant population is very young and in early expansive phase^18^,^25^,^26^, while the other two populations of *X. securis* analyzed here were reported from the respective regions at least one decade ago^53^,^54^. In the expansive phase of the invasion, many genes might be activated for maximizing the ability of invaders to adapt and effectively utilize the resources available in the new habitat. Therefore, the invasive success of a species would benefit from relaxed gene expression control -low levels of DNA methylation would increase transcriptional opportunities and enhance activity of transposable elements^55^. However as long as the invasion process advances, global methylation would acquire normal levels, allowing intrinsic regulation mechanisms, control of population growth and exhibiting common response to environmental stress. In our study *F. enigmaticus*, especially the older introduced population in Bay of Biscay –although not the more recent introduction in New Zealand, could be an example. Epigenetic variation may thus be linked to the ‘age’ of the introduced population^13^,^14^,^15^ or the phase of an invasion. Although this should be confirmed from further studies, perhaps increased epigenetic polymorphism or decreased global methylation in a newly arrived population could serve as an indicator of invasiveness - and thus a proxy of its threat to local ecosystems.

Another interesting finding in our study was significantly higher epigenetic than genetic diversity in both highly invasive species, *X. securis* and *F. enigmaticus*. Not a minor discovery, we provided here the first evidence of methylation in the two studied species. To date, regulation of gene expression through DNA methylation has been described for other molluscs^55^,^56^ and polychaetes^6^, but not for these particular species. The methylation patterns described in those earlier studies were generally associated with ecological contexts^56^. In our results, despite the large geographical scope of this study involving populations from distant areas, the genetic variation (NML and total AFLP) in *F. enigmaticus* from the three analyzed populations did not differ much. It differed more among *X. securis* samples. Still, genetic diversity was clearly and significantly lower than epigenetic diversity in both species. Epigenetic differences with similar genetic variation were reported in a few studies of introduced populations of plants and vertebrates^13^,^14^,^15^. The present findings from six marine invertebrate populations would also support the idea of changes in methylation being involved in a general mechanism of biological invasions independently from genetic variation.

This proof-of-concept study could open new perspectives for better understanding marine biological invasions, their ecology and intrinsic mechanisms. The epigenome analysis of donor and introduced populations along with the population status assessment (i.e. established, expanding, outbreak, accommodated) is highly desirable. Discovering the key genes involved in adaptation mechanisms of marine invaders, as well as their epigenetics would provide useful information for prioritizing risks associated with particular invasive species and undertaking adequate response measures.

## Additional Information

**How to cite this article:** Ardura, A. *et al*. Epigenetic signatures of invasive status in populations of marine invertebrates. *Sci. Rep.* **7**, 42193; doi: 10.1038/srep42193 (2017).

**Publisher's note:** Springer Nature remains neutral with regard to jurisdictional claims in published maps and institutional affiliations.

## Supplementary Material

Supplementary Data 1

Supplementary Data 2

## Figures and Tables

**Table 1:** Generalized environmental conditions at the sampling (specimen collection) sites.

Sample code	Country	Region	First report	Location	Coordinates	Temperature	Sunlight	Rainfall	Habitat	Salinity	Pollution
***Xenostrobus securis***											
XCant	Spain	South Bay of Biscay	2014^26^	Aviles estuary	43° 33′ 22″N, 5° 55′ 20″W	9.6 (6.8–19.8)	1,670	1,048	Marina, international port	29.3 (17.9–35.8)	Urban, industrial
XAtl	Spain	Northwest Spain	2002^53^	Pontevedra estuary	42° 26′ 7.6″N, −8° 38′ 56″W	14.8 (10.4–19.2)	2,247	1,613	Marina, international port	33 (31.5–35)^57^	Urban, industrial
XMed	France	Mediterranean Sea	1992^54^	Vidourle Lagoon	43°34′42.14″N 4°02′34.98″E	15.1 (10.4–19.9)	2,668.2	629.1	Lagoon/Natura 2000 Network	23.9 (20.4–27.3)^58^	Urban, eutrophication
***Ficopomatus enigmaticus***											
FCant	Spain	South Bay of Biscay	1920s^30^	Llanes	43°25′16″N, 4°45′11″W	13.5 (9.9–17.1)	1,756	1,062	Marina, small fishing port	25.2 (21.4–29.5)	Urban, small village
FMed	France	Mediterranean Sea	1947^59^	Saint-Nazaire Lagoon	42°38′49.25″ N 3°01′27.75″ E	15.7 (11.4–20.1)	2,464.9	557.6	Lagoon/Natura 2000 Network	20.4 (5.55–35.2)^60^	Urban, eutrophication^61^
FNZ	New Zealand	South Pacific Ocean	1967^62^	Napier, Hawke’s Bay	39°29′S 176°55′E	16.4 (12.2–22.1)	2,281	809.7	Marina, international port	34–35^63^	Urban, industrial

In the sample code ‘X’ refers to *X. securis* samples and ‘F’ to *F. enigmaticus* samples. International ports receive >one million annual cargo tons. Temperature, average annual temperature in °C (min-max); sunlight, average sunlight hours per year; rainfall, annual rainfall in mm; salinity in ppm (min-max).

**Table 2:** Statistical analysis of individual methylated loci for *X. securis* (above) and *F. enigmaticus* (below). ANOVA or Welch F test, and Kruskal-Wallis H analysis, for comparing means and medians respectively; Tukey’s and Mann-Whitney post-hoc tests for respective pairwise comparisons of means and medians (in italics, statistically significant p-values).

Xenostrobus securis								
	Test for equal means: ANOVA				Test for equal medians			
	Sum of squares	df	Mean square	F	p (same)	H (chi2):	28.71	
Between groups	0.27815	2	0.1391	18.34	0.0000003	Hc (tie corrected):	28.72	
Within groups	0.599038	79	0.0076			p (same):	0.0000006	
Total:	0.877188	81						
Tukey’s post-hoc					**Mann-Whitney post-hoc (P-values)**			
	Atlantic	Bay of Biscay	Mediterranean					
Atlantic		0.0001154	0.9968				Atlantic	Bay of Biscay
Bay of Biscay	7.126		0.0001135			Bay of Biscay	0.000003	
Mediterranean	0.1094	7.235				Mediterranean	0.6993	0.000009
***Ficopomatus enigmaticus***								
**Test for equal means: Welch F**						**Test for equal medians**		
F	df	P-value				H (chi2):	5.41	
4.969	6, 186	0.051				Hc (tie corrected):	5.41	
						p (same):	0.066	
**Tukey’s post-hoc**								
	Bay of Biscay	Mediterranean	New Zealand			**Mann-Whitney post-hoc (P-values)**		
Bay of Biscay		0.08647	0.1012				Bay of Biscay	Mediterranean
Mediterranean	3.4		0.9947			Mediterranean	0.06619	
Nwe Zealand	3.26	0.1401				New Zealand	0.0606	0.9025

**Table 3:** Pairwise differentiation between sampled populations measured as ɸ_ST_ (P-value in parenthesis), for methylation sensitive or epigenetic loci (below diagonal) and non-methylated or genetic loci (above diagonal).

	XAtl	XMed	XCant	FNZ	FCant	FMed
XAtl		0.016 (P = 0.004)	0.139 (p < 0.0001)	—	—	—
XMed	0.012 (P = 0.157)		0.132 (p < 0.0001)	—	—	—
XCant	0.165 (p < 0.0001)	0.182 (p < 0.0001)		—	—	—
FNZ	—	—	—		0.0203 (P = 0.387)	0.067 (P = 0.168)
FCant	—	—	—	0.187 (P = 0.009)		−0.015 (P = 0.535)
FMed	—	—	—	0.205 (P = 0.018)	0.021 (P = 0.343)	

F and X letters represent *F. enigmaticus* and *X. securis* respectively, and Atl, Cant, Med and NZ correspond accordingly to Atlantic, Bay of Biscay, Mediterranean and New Zealand populations.
